# Centralized Multi-Hop Routing Based on Multi-Start Minimum Spanning Forest Algorithm in the Wireless Sensor Networks

**DOI:** 10.3390/s21051775

**Published:** 2021-03-04

**Authors:** Rencheng Jin, Xiaolei Fan, Ting Sun

**Affiliations:** Key Laboratory for Micro/Nano Technology and System of Liaoning Province, Dalian University of Technology, Dalian 116024, Liaoning, China; f2019@mail.dlut.edu.cn (X.F.); suntingln@mail.dlut.edu.cn (T.S.)

**Keywords:** bottom-up, clustering, multi-hop, multi-start minimum spanning forest, routing

## Abstract

Wireless sensor networks (WSNs) are widely applied in environmental monitoring, target tracking, military, and industrial fields. However, the battery energy of sensor nodes in WSNs is limited, which limits its development. Previous studies have shown that clustering protocols and multi-hop communication are beneficial to reduce nodes energy consumption. The multi-hop protocol based on low energy adaptive clustering hierarchy (LEACH) has been proven to significantly reduce energy dissipation. However, LEACH-based multi-hop protocols generally have the problem of unbalanced energy dissipation and data conflicts. In this paper, we propose a centralized multi-hop routing based on multi-start minimum spanning forest (LEACH-CMF) to optimize LEACH. In order to realize multi-hop communication, we introduced a multi-start minimum spanning tree algorithm to select relay nodes with the minimum relay cost and generate appropriate multi-hop paths. To avoid data collision in multi-hop communication and make nodes including the cluster heads sleep as much as possible in the non-working state, we design a bottom-up continuous time slot allocation method to improve the time division multiple access (TDMA) cycle. We performed simulation in NS2. The simulation results show that the network lifetime is approximately doubled compared to LEACH and centralized low energy adaptive clustering hierarchy (LEACH-C). The simulation results show that the proposed protocol can effectively balance the energy dissipation of nodes and prolong network lifetime.

## 1. Introduction

Wireless sensor networks (WSNs) are composed of large numbers of nodes (few tens to thousands), which have characteristics of self-organizing, low cost, and random deployment [1]. Sensor nodes are randomly distributed in the monitoring area and sense signals in the surrounding environment through built-in sensors, then send the collected data to base station (BS). They have been widely applied in environmental monitoring [2], forest fire detection [3], military [4], precision agriculture [5], industry, etc., and thus have attracted the interest of researchers in recent years [6,7]. However, an important factor affecting the application of WSNs is limited energy. Sensor nodes usually carry small batteries and it is hard to recharge or replace batteries in complex environments [8]. Therefore, in practical applications, each node in the WSNs is required to consume energy as little as possible for data collection and transmission to prolong network lifetime. In order to save energy and maximize network lifetime as much as possible, routing protocols should be more energy efficient. Therefore, reducing node energy consumption has become the main goal of routing protocols.

In WSNs routing protocols, the clustering routing protocol has a great improvement in energy consumption compared to other routing protocols. In clustering routing, WSNs is divided into several clusters, each cluster is composed of a cluster head and some common nodes. Cluster heads can form a higher level network until the highest-level BS. Low energy adaptive clustering hierarchy (LEACH) [9] is a typical clustering routing. LEACH uses a cluster election algorithm to divide the WSNs into several clusters. Common nodes in the cluster send the collected data to the cluster head, then the cluster head sends data to BS after data aggregation. LEACH uses a threshold formula to select cluster heads in each round, which effectively avoids the excessive energy consumption of cluster heads. In addition, data aggregation can reduce the amount of communication, thus prolong network lifetime. Centralized low energy adaptive clustering hierarchy (LEACH-C) [10] is an improved centralized routing protocol based on LEACH. The difference from the LEACH is that the cluster heads selection is the responsibility of the BS, which selects a group of nodes with higher density as optimized cluster heads by simulated annealing algorithm. There are other clustering routing protocols, such as PEGASIS [11], HEED [12], and other optimized protocols [13,14,15]. However, in the clustering routing, the cluster heads need to be responsible for data collection, data aggregation, and transmission, which consume more energy than common nodes, resulting in unbalanced energy dissipation.

Another primary technique is multi-hop routing, which is a very efficient way to relay packets in the network. In the multi-hop routing protocol, an algorithm is introduced to construct a tree topology, with BS as the root node. Multi-hop routing reduces energy consumption in WSNs [16]. Common nodes send data to intermediate nodes, and intermediate nodes perform data aggregation and then send data to the next intermediate node until the data is sent to BS. LEACH-based multi-hop protocols include inter-cluster multi-hop protocols, intracluster multi-hop protocols, and combination of intra- and intercluster protocols. There are some multi-hop protocols based on LEACH, such as M-LEFCA [17], DMH-LEACH [18], EM-LEACH [19], LEACH-WM [20], and more [21,22,23]. However LEACH-based multi-hop protocols generally have the problem of unbalanced energy dissipation and data conflicts.

In this paper, a centralized multi-hop routing protocol LEACH-CMF is proposed, which is based on the multi-start minimum spanning forest algorithm to realize multi-hop communication. The multi-start minimum spanning forest algorithm can select relay nodes with the minimum relay cost and generate appropriate multi-hop paths. We use multi-hop communication to shorten the communication distance between most nodes. In order to solve the problem of data conflicts that may be caused by multi-hop communication, we design a bottom-up continuous slot time allocation strategy. Each node sends data in the assigned time slot and sleeps as soon as possible. The proposed method balances the load of the network, has lower energy dissipation and better energy dissipation stability, and thus prolongs network lifetime.

The rest of this paper is organized as follows. Section 2 introduces related works and analyzes the exiting problems. Section 3 explains the network system model. Section 4 introduces our proposed method in detail, including multi-start minimum spanning tree algorithm and the improvement of time division multiple access (TDMA). In Section 5, we conducted simulation experiments for the protocol we proposed and compared the simulation results. Finally, we made a conclusion based on the experimental results.

## 2. Related Works

**Figure 1:**
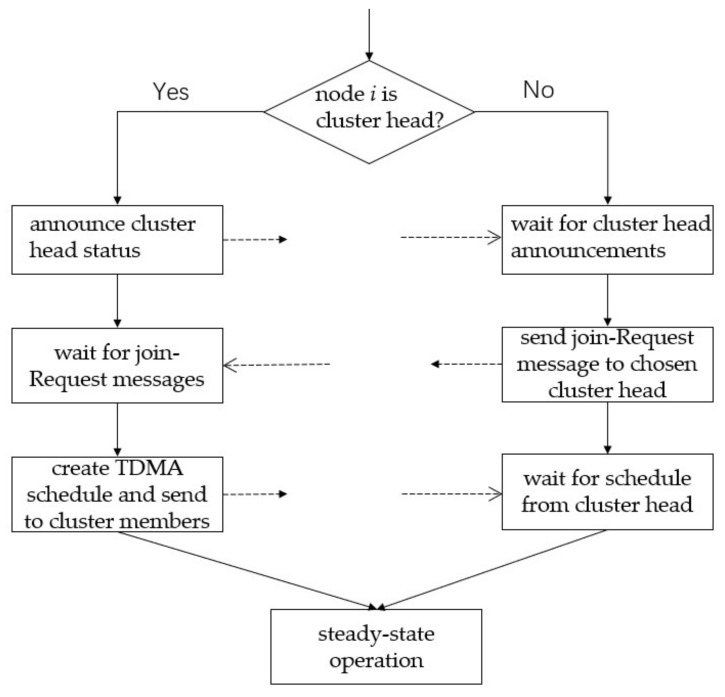
The step of low energy adaptive clustering hierarchy (LEACH).

The LEACH protocol is completely a clustering routing protocol. Clustering is the most common technique to balance energy consumption among nodes, while minimizing traffic and overhead during the data transmission phases [24]. The lifetime of LEACH consists of many rounds, and each round consists of a set-up phase and steady-state phase. During the set-up phase, all nodes select a random number between 0 and 1. If the random number is less than a threshold T(n), the node becomes a cluster head. The cluster head broadcasts cluster head message, and other nodes choose the nearest cluster head and become a member of the cluster. The threshold T(n) is set as:

(1) $$T(n)=\left\{\begin{array}{l} \frac{p}{1-p(r\mathrm{mod}1/p)}n\in G\\ 0\;\;\;\;otherwise \end{array}\right.$$

where *p* is the desired cluster head ratio, *r* is the current number of rounds, and *G* is the set of nodes that have not served as a cluster head in the recent 1/*p* round. In the steady-state phase, TDMA is used in the cluster to avoid data conflicts, and CDMA is used between clusters to avoid transmission conflicts between clusters [25]. The cluster heads create a TDMA schedule and allocate time slots to each common node. Common nodes transmit data to cluster heads in their allocated transmission time slot. Cluster heads collect and fuse the data from common nodes and send the fused data to BS. In this way, LEACH can reduce network energy dissipation and prolong network lifetime. Figure 1 shows the steps of LEACH. LEACH-C [10] uses a centralized clustering algorithm and the same steady-state protocol as LEACH. During the set-up phase, each node sends information about its location and residual energy to BS. BS calculates the average energy of the nodes, and the node whose remaining energy is lower than the average energy cannot be selected as a cluster head. BS runs the simulated annealing algorithm to determine the cluster heads in each round. The simulated annealing algorithm minimizes the total sum of squared distances between common nodes and the cluster heads to reduce energy dissipation.

LEACH reduces energy consumption through clustering, but the cluster heads are responsible for data aggregation and transmission, which lead to accelerated death of cluster heads and cause unbalanced network load. In recent years, many researchers have combined multi-hop communication with LEACH. Most of the multi-hop protocols improved for LEACH mainly focus on intercluster multi-hop. Barzin et al. [24] proposed a multiobjective nature-inspired algorithm based on the shuffled frog-leaping algorithm and firefly algorithm (MOSFA) as an adaptive application-specific clustering-based multi-hop routing protocol. MOSFA’s multiobjective function regards different criteria (inter- and intracluster distances, the residual energy of nodes, distances from the sink, overlap, and load of clusters) to select appropriate cluster heads at each round. A backward-directed backbone (BDB) is used to route the gathered data from the selected clustering heads (CHs) to the sink by multi-hop routing. CHs select the most appropriate node as its forwarder relay node using the multiobjective function. The lower-level cluster head sends data to the higher-level cluster heads. Toor et al. [26] proposed an energy aware cluster based multi-hop (EACBM) protocol. Sensor nodes in the network are heterogeneous nodes, which have three different types (normal, intermediate, and advance SNs) of energy level. A new probabilistic equation is used to select cluster heads. There are always some sensor nodes that have not joined any clusters, because they are far away from the cluster head range. In order to cover those sensor nodes a subcluster and a subcluster head is created. A multi-hop intercluster can reduce the transmission distance, reduce the energy consumption of cluster heads, and effectively prolong network lifetime.

The introduction of multi-hop intracluster can easily cause data conflicts and most protocols did not consider how to solve this problem. Zhang et al. [20] proposed a new energy-efficient algorithm (LEACH-WM) based on the weight and intracluster multi-hop mechanism. In the steady phase of LEACH-WM, weight relay nodes are selected in the cluster according to common nodes’ residual energy and their distances away from BS. Common nodes send data to the cluster head in the cluster. Cluster head sends aggregation data to its weight relay, which sends data to the BS. This protocol decreases energy dissipation of the cluster heads and distributes energy dissipation more uniformly in the network and further prolongs network lifetime. Kirsan et al. [27] propose a multi-hop simulated annealing (MhSA-LEACH) algorithm based on intracluster multi-hop communication. The selection of the cluster head adopts the simulated annealing algorithm, which takes into account the remaining energy of the nodes. Relay nodes in multi-hop path are selected using the simulated annealing (SA) algorithm for the traveling salesman problem (TSP), which can find the optimal path for data transmission. The intracluster multi-hop data transmission path is chained, and the cluster head is also the chain head. This protocol reduces the communication distance of nodes within the cluster through multi-hop to reduce energy consumption.

Introducing inter- and intracluster multi-hop communication in large-scale networks can shorten the communication distance and effectively improve energy efficiency. However, there are not so many protocols that simultaneously introduce inter- and intracluster multi-hop routing. The protocol proposed by Siavoshi et al. [28] introduces a geographical multilayered clustering where the network is divided into three layers. The size of clusters increases or decreases with the distance from the BS. In each cluster, using the remaining energy of the parent node and the number of neighboring parent nodes as input parameters, one or two parent nodes are selected using fuzzy logic algorithm. A subtree topology is formed to balance energy consumption in the whole network. Parent nodes as relay nodes are responsible for collecting and compressing the data from the child nodes. This protocol also improves cluster heads election formula used in LEACH and takes into account the remaining energy of the nodes, which prevents nodes with lower energy from being selected as the cluster head and decreases the packet loss rate. This protocol decreases inter- and intracluster communication energy costs. Ammar et al. [29] proposed a novel multi-hop clustering cross layer protocol. This protocol proposed an algorithm to determine whether the source nodes need to send its data directly to the BS or use a relay node. The algorithm takes into account the remaining energy of nodes, link quality and physical distance between nodes when creating multi-hop routing path. The introduction of multi-hops communications has considerably improved LEACH by finding the best path between the source cluster heads and the destination.

The introduction of multi-hop communication in the clustering protocol can shorten the communication distance of nodes, reduce the energy consumption and effectively prolong network lifetime. However, most LEACH-based multi-hop routing protocols use simplified protocol models and simulation methods, ignoring several practical issues:

(1) Studies focus on cluster head selection and intercluster multi-hop transmission are popular. Rarely, network load is balanced by optimizing intracluster data transmission. Cluster heads still bear a large load and are the main nodes of energy dissipation.
(2) Direct introduction of multi-hop mechanism in the LEACH protocol will cause data conflicts. However, none of them proposed corresponding solutions to solve this problem.

## 3. Network Model

### 3.1. Model Assumptions

Our proposed protocol is based on the following model assumptions:

(1) All nodes are randomly distributed in the monitoring area and the position coordinates are known. At the beginning of each round, a data packet with their own energy and position information is sent to the BS.
(2) The nodes are homogeneous, with the same initial energy, and have data aggregation capability.
(3) All nodes are static and do not move during communication.

### 3.2. Energy Dissipation Model

This paper used the same energy dissipation model as in [9]. When a node transmits a *k*-bit data packet to a location with a distance of *d*, the energy consumed is composed of the transmission circuit loss and the power amplification loss as:

(2) $$E_{TX}=\left\{\begin{array}{l} E_{elec}\times k+\varepsilon_{fs}\times k\times d^{2}\;d<d_{0}\\ E_{elec}\times k+\varepsilon_{mp}\times k\times d^{4}\;d\geq d_{0} \end{array}\right.$$

when the node receives a *k*-bit data packet, the energy consumed is the loss of the transmitting circuit as:

(3) $$E_{RX}=E_{elec}\times k$$

nodes fuse *k*-bit data, and the energy consumed is

(4) $$E_{DA}=E_{da}\times k$$

where $E_{elec}$ is the circuit energy dissipation coefficient, $\varepsilon_{fs}$ is the free space amplification coefficient, $\varepsilon_{mp}$ is the multipath fading amplification coefficient, $E_{da}$ is the data aggregation energy dissipation coefficient, *d* is the transmission distance, and *d*_0_ is the transmission distance threshold and is obtained by the following equation

(5) $$d_{0}=\sqrt{\frac{\varepsilon_{fs}}{\varepsilon_{mp}}}$$

Based on the above analysis, we conclude that the transmission distance should be reduced as much as possible to reduce energy consumption to send more data.

### 3.3. TDMA Schedule

In the set-up phase, cluster head creates a TDMA schedule based on the number of nodes in the cluster. Each node sends data in the time slot specified in the TDMA schedule to avoid data conflicts. By analyzing the MIT LEACH protocol source code in NS2 simulation software, we can find that the time of a frame is set as

(6) $$T_{frame}=(n+5)\times T_{slot}$$

where $T_{slot}$ is the time of a slot, which represents the time required for data transmission. *n* is the number of common nodes in the cluster, and 5 is a redundant value to ensure that the cluster head can complete a data transmission to the BS. $T_{frame}$ is a frame time, which represents the time for all nodes in the same cluster to complete a data transmission. Different clusters have different frame time. The time of a round is set as

(7) $$T_{round}=T_{setup}+x\times T_{frame}$$

**Figure 2:**
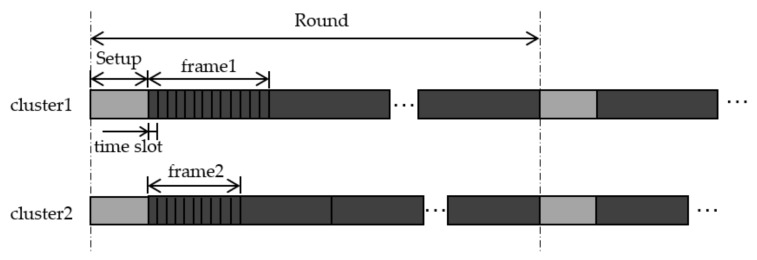
The TDMA schedule of two clusters. Cluster1 is a larger cluster with more nodes than cluster2. The gray grids represent the set-up phase, and the black grids represent the steady-state phase.

$T_{round}$ is the time of a round, a given priori value. $T_{setup}$ is the time of the set-up phase, also a given value. *x* is the number of frames in a round, that is to say, the steady-state phase consisted of *x* frames. Since the size of each cluster was different, the TDMA cycle of each cluster was different, and the number of frame *x* was also different. The round and TDMA schedule of two clusters are shown in Figure 2.

With the introduction of multi-hop transmission, the time of data transmission between cluster heads will collide with the time of data collection by cluster heads. The time collision will lead to data loss. Since though the TDMA is used, each cluster head should listen to only one code and cannot receive different data packets with the same code in the same time. Especially, when a cluster head of a smaller cluster (cluster2) sends data to a cluster head of a larger cluster (cluster1), cluster2’s cluster head completes the data collection within the cluster2 first. Cluster2’s cluster head may send date to cluster1’s cluster head when cluster1’s cluster head is receiving data from cluster1’s member nodes. Data conflict will occur at this time. Moreover, because there is *x* frames for the cluster2, the cluster2 will send data to the cluster1 at a higher frequency than the cluster2 send data to the next cluster head or BS, which will easily cause network congestion and data loss, and the simulation will not continue. In addition, because the transmission frequencies of the cluster heads do not match, the cluster heads must keep awake at all times and are unable to properly sleep. Therefore, it is necessary to improve TDMA to adapt to multi-hop communication.

## 4. Proposed Method

### 4.1. Analysis of Intracluster Relay Energy

In order to realize the intracluster multi-hop relay, we first analyzed the intracluster relay cost.

**Figure 3:**
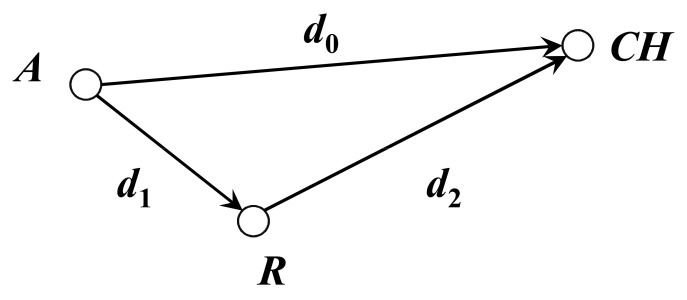
Node relays in a cluster.

As shown in Figure 3, suppose node *A* and *R* are nodes in the cluster, and the cluster head is node *C*. In non-relay case, node *A* and *R* send *k*-bit data to *C* respectively, the total energy dissipation is

(8) $$E_{1}=k\times (E_{elec}+\varepsilon_{fs}\times d_{0}^{2})+k\times (E_{elec}+\varepsilon_{fs}\times d_{2}^{2})+2\times k\times E_{elec}$$

In the case of relay, node *A* sends data to node *R* firstly. Node *R* can fuse the data and then send data to node *C*. The total energy dissipation is

(9) $$E_{2}=k\times (E_{elec}+\varepsilon_{fs}\times d_{1}^{2})+k\times (E_{elec}+\varepsilon_{fs}\times d_{2}^{2})+2\times k\times E_{elec}+2\times k\times E_{da}$$

when *E*_2_ < *E*_1_, the result can be simplified as

(10) $$\varepsilon_{fs}\times d_{1}^{2}+2\times E_{da}<\varepsilon_{fs}\times d_{0}^{2}$$

According to the above analysis, the energy cost of the relay case will be smaller than the energy cost of the non-relay case when the condition of Equation (10) is satisfied. Each node in the cluster can select an appropriate node as a relay node, and each relay node can perform a data aggregation after receiving all data packets from the nodes it relays. So, the remaining energy of the relay nodes should be as much as possible. The merged data is then forwarded to the relay node’s relay node or BS. Through such a relay mechanism, the energy efficiency of the whole network will be improved.

Based on the above analysis, to select a suitable relay for a node in the cluster, the relay cost is defined as follows:

(11) $$Cost=\left\{\begin{array}{cc} \frac{\varepsilon_{fs}\times d_{1}^{2}+2\cdot E_{da}}{\varepsilon_{fs}\times d_{0}^{2}}\times \frac{E_{avg}}{E_{rest}} & E_{rest}\geq E_{avg}\\ +\infty & E_{rest}<E_{avg} \end{array}\right.$$

where *E_rest_* is the residual energy of the candidate relay node and *E_avg_* is the average residual energy of all nodes in the network and is calculated as follows:

(12) $$E_{avg}=\frac{1}{N}\sum_{i=1}^{N}E_{i}$$

According to Equation (11), when *d_1_* decreases and *d_0_* increases, the cost of relay will decrease. Due to a higher data pressure than the non-relay node, the relay node may die prematurely, leading to link interruption. Therefore, the *E_avg_*/*E_rest_* is used as the energy coefficient to select nodes with high residual energy. For non-cluster head nodes in any cluster, the node with the lowest relay cost is selected as the relay node according to the relay cost Equation (11). The node with residual energy more than the average energy of the network was selected as the candidate relay node. The smaller the cost, the more likely for a node to be selected as a relay node.

### 4.2. Analysis of Intercluster Relay Energy

In order to realize the intercluster multi-hop relay, we first analyzed the intercluster relay cost.

As shown in Figure 3, suppose node *A* and *R* are cluster heads and node *C* is the BS. In non-relay case, cluster head *A* and *R* send *k*-bit data to *C* respectively, the total energy dissipation is

(13) $$E_{1}=k\times (E_{elec}+\varepsilon_{fs}\times d_{0}^{2})+k\times (E_{elec}+\varepsilon_{fs}\times d_{2}^{2})$$

In the case of relay, cluster head *A* first sends data to cluster head *R*. cluster head *R* canfuse the data and then sends data to BS *C*. The total energy dissipation is

(14) $$E_{2}=k\times (E_{elec}+\varepsilon_{fs}\times d_{1}^{2})+k\times (E_{elec}+\varepsilon_{fs}\times d_{2}^{2})+k\times E_{elec}+2\times k\times E_{da}$$

when *E*_2_ < *E*_1_, the result can be simplified as

(15) $$\varepsilon_{fs}\times d_{1}^{2}+2\times E_{da}+E_{elec}<\varepsilon_{fs}\times d_{0}^{2}$$

To select a suitable relay for a cluster head, the relay cost is defined as follows:

(16) $$Cost=\left\{\begin{array}{l} \frac{\varepsilon_{fs}\times d_{1}^{2}+2\times E_{da}+E_{elec}}{\varepsilon_{fs}\times d_{0}^{2}}\times \frac{E_{avg}}{E_{rest}}E_{rest}\geq E_{avg}\\ +\infty \;\;\;\;\;\;\;E_{rest}<E_{avg} \end{array}\right.$$

where *E_rest_* is the residual energy of the candidate relay node and *E_avg_* is the average residual energy of all cluster heads in the network and is calculated as Equation (12). For a cluster head, the candidate cluster head with the lowest relay cost was selected as the relay node according to the relay cost Equation (16). The smaller the cost, the more likely for a cluster head to be selected as a relay cluster head node. Multi-hop transmission between clusters can reduce the load of the cluster heads and thus balance the network load.

### 4.3. Multi-Start Minimum Spanning Forest Algorithm

This protocol uses the same method as LEACH-C to select a group of cluster heads. After selecting the cluster heads, the relay nodes of each non-cluster head node should be selected. In order to minimize the relay cost between each node and its relay node in the network, a multi-start minimum spanning forest algorithm is proposed. This algorithm first takes *k* cluster heads as *k* starting root nodes, and then calculates the relay cost by Equation (11) as the weight of the directed edge to generate k non-intersecting minimum spanning trees simultaneously in the network. The multi-start minimum spanning forest consists of multiple minimum spanning trees, and its root is the base station. The roots of these minimum spanning trees are the cluster heads, and each edge has the smallest weight. This is the process of generating multi-hop paths in the cluster.

Assume that the total number of nodes is *N* and the number of clusters is *k*, *ch[i]* (*i* = 1, 2, …, *k*) represents the *i*th cluster head node. The network is represented by a connected network with directed weights. According to the graph theory, the entire network can be expressed as

(17) P=(V,E)

where *V* is the set of all nodes in the network and *E* is the set of all directed edges in the network.

*TU[i]* (*i* = 1, 2, …, *k*) is the set of nodes in the *i*th minimum spanning tree, *TE[i]* (*i* = 1, 2, …, *k*) is the set of edges in the *i*th minimum spanning tree, and *U = TU[1]∪TU[2]∪⋯∪TU[k]* is the set of all nodes in the current whole *k* minimum spanning trees. <*u*, *v*> is a directed edge and the weight represents the relay cost when node *v* chooses node *u* as a relay.

The algorithm construction process as follows:

(1) Initialize the *TU[i]* and *TE[i]*, *TU[i] = {ch[i]}, TE[i]* = {}, (*i* = 1, 2, …, *k*).
(2) Find the directed edge <*u_0_, v_0_*> with the smallest weight among the edges <*u*, *v*> where *u* ∈ *U*, *v* ∈ *V−U*. If *u_0_* ∈ *TU[j] (*1 *≤ j ≤ k)*, add the edge (*u_0_*, *v_0_*) to the set *TE[j]*, and add the node *v_0_* to the set *U* at the same time. Then, *u_0_* is selected as a relay node of node *v_0_*, and *u_0_*’s cluster head *ch[i]* is set as the cluster head of node *v_0_*.
(3) Repeat step 2 until *U = V*. Finally, each node selects a relay node and a cluster head except for the cluster head node.

**Figure 4:**
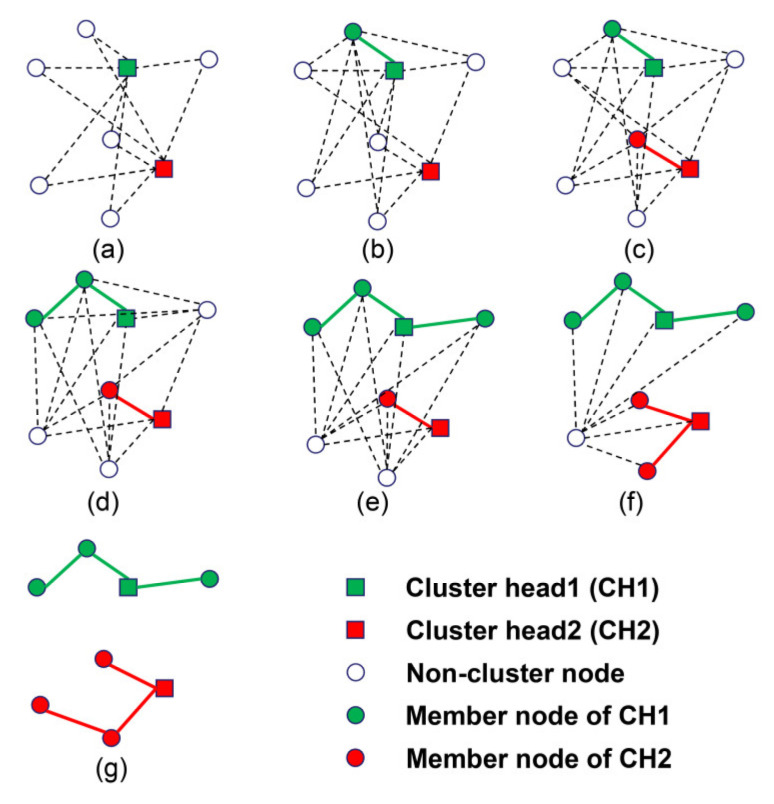
(**a**–**g**) Process of multi-start minimum spanning forest algorithm with two clusters.

The clustering process of the multi-start minimum spanning forest algorithm is shown in Figure 4. In the figure, squares represent cluster heads, hollow circles represent nodes, which have not joined a cluster, and solid circles represent nodes that have already joined a cluster. There are two starting points at the beginning as Figure 4a. By comparing the relay costs, each time a node, which has entered a cluster selects a node, which has never entered the cluster as its relay node and joins the cluster as Figure 4b–g. Six times later, all six non-cluster head nodes join the clusters and two disjoint clusters are formed as Figure 4g.

**Figure 5:**
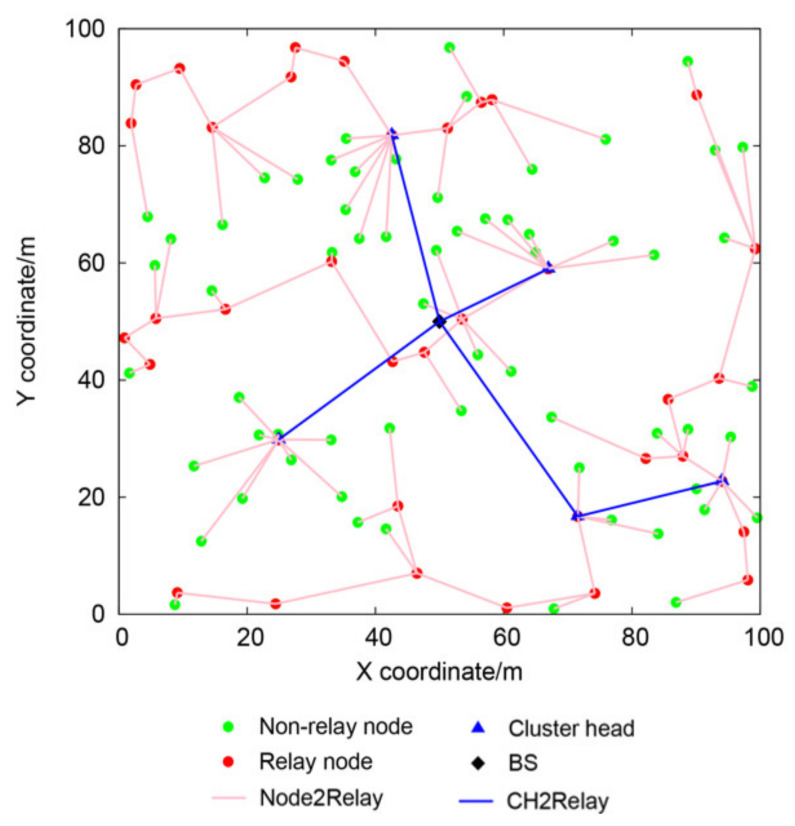
The topology of the network.

In the phase of multi-hop transmission between clusters, with the BS as the only starting point and the cost of Equation (16) as the edge weights, a tree network between the cluster heads can be generated. The overall topology of the network is shown in Figure 5. 

### 4.4. Time Slot Division Based on TDMA

The intracluster data transmission follows the time division multiplexing (TDMA) [12] method of the LEACH protocol. The relay node must receive data in the wake state. If the node acting as a relay node remains awake at all times, it will cause additional energy dissipation. So, the relay node needs to wake up at the beginning of each relay node’s time slot of sending data, and then sleep after receiving the data. A node acting as a relay node may need to receive data from multiple nodes, which will cause the node to constantly switch between the awake state and the sleep state.

In practice, turning off and turning on the wireless transceiver takes time, and constantly turning on and off the network transceiver is not conducive to obtaining a stable signal. In order to suppress this situation, this paper designs a bottom-to-up continuous time slot allocation method, that is, to allocate time slots to each layer in a bottom-up order, and assign consecutive time slots to nodes at the same layer. As we all know, breadth-first traversal can visit all nodes in the tree structure once, and only once. This just meets the needs of time slot allocation.

**Figure 6:**
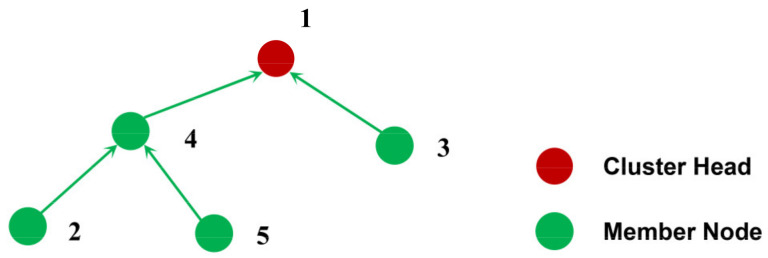
A simple cluster.

As shown in Figure 6, a cluster contains five nodes, of which node 1 is the cluster head, node 3 directly sends data to the cluster head, and nodes 2 and 5 need to relay through node 4 to forward data to the cluster head.

Let {2, 5} be the first layer node and {4, 3} be the second layer node. First allocate time slots to nodes {2, 5}, and then allocate time slots to {4, 3}.

The appropriate allocation results are:

1) 2→5→3→4→1; 2) 2→5→4→3→1; 3) 5→2→3→4→1; 4) 5→2→4→3→1.

In order to implement the above allocation strategy, we first built a logical tree structure with the cluster head as the root node according to the relay relationship, and then traversed the tree to obtain a traversal list by breadth-first algorithm. Finally, we could arrange the traversal list in reverse order to get a TDMA scheduling table.

After that, we assigned data transmission wake-up time slots to each node in the order of the nodes in the TDMA scheduling list. Then, each node was also allocated a data reception wake-up time slot, which corresponds to the time slot in which the first data to be received by the node was transmitted. Each node wakes up in the data reception wake-up time slot, starts to continuously receive data, and enters sleep after receiving all data. In the data transmission wake-up time slot, the node wakes up again, performs data aggregation, sends the fused data to the next relay node, and then sleeps again.

In order to match the sending frequency between different cluster heads at the lowest delay, avoid the data collision, and allow the cluster heads to sleep properly in the non-working phase, this paper further improved the TDMA cycle based on the TDMA division above.

**Figure 7:**
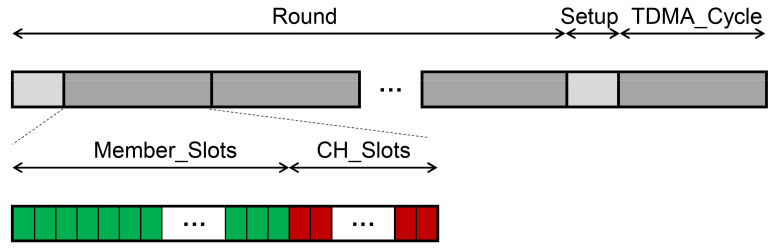
The improved TDMA schedule. The gray grids represent the set-up phase, and the black grids represent the steady-state phase. The green grids indicate the time slots of the member nodes in the cluster, and the red grids indicate the time slots of other cluster heads.

A TDMA cycle was divided into two stages: the stage of data collection within cluster and the stage of data transmission between cluster heads. During the data collection stage, the nodes in the cluster wake up to accept data from nodes they relay and send data to the selected relay node at the allocated time slot. During the data transmission phase between cluster heads, the cluster head also receives the data of the previous cluster heads and sends the data to the next-hop relay node in the assigned time slots. Since the cluster heads and the BS also form a tree-like topology structure, this paper also divided the time slots for data transmission between cluster heads according to the bottom-up continuous time slot allocation method. The time slots distribution of a frame is shown in Figure 7 and Equations (18) to (21).

(18) $$m=\max(n_{i})i=1,\;2,\;\ldots ,\;k$$

(19) $$T_{member}=m\times T_{slot}$$

(20) $$T_{chs}=c\times T_{slot}$$

(21) $$T_{frame}=T_{member}+T_{chs}$$

where *n_i_* is the number of nodes of cluster *i*, *m* is the max cluster size or the number of nodes in the cluster with the most nodes, *c* is the total number of cluster heads, *T_member_* is time of the stage of data collection within cluster, and *T_chs_* is the time of the data transmission stage between cluster heads.

### 4.5. Overall Protocol Flow

**Figure 8:**
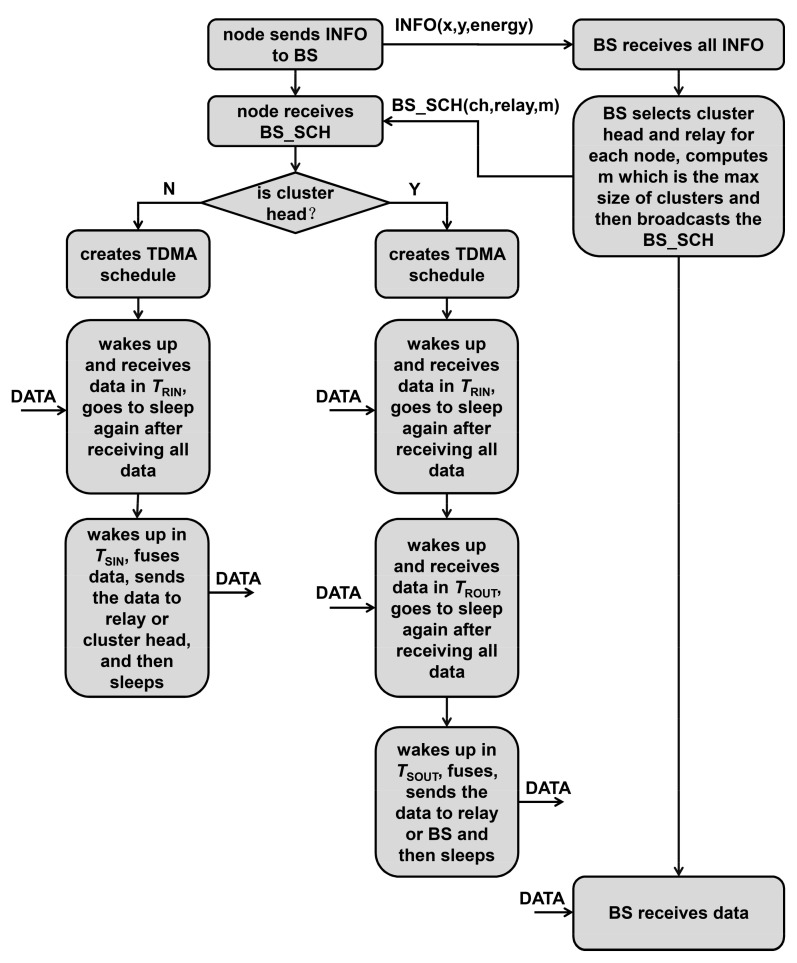
The protocol flow.

The specific protocol flow is shown in Figure 8 and described as follows:

Step 1: Node broadcast. Each node sends an INFO message with its own information to the BS, which contains the coordinates of the node and the residual energy of the node.

Step 2: After receiving all broadcast information, the BS first selects a group of optimized cluster heads according to the simulated annealing algorithm, then selects the cluster head node and the relay node for each node according to the multi-start minimum spanning forest algorithm, and finally broadcasts the BS_SCH message, which consists of the cluster head id of each node, the relay node id of each node and the max cluster size. Relay nodes of common nodes in the cluster are other common nodes or cluster heads in the cluster, and the relay nodes of the cluster head are other cluster heads or the BS.

Step 3: Each node receives the BS_SCH message from the BS and creates an intracluster TDMA scheduling sequence. For each non-cluster head node, it will calculate the intracluster data reception wake-up time slot *T_RIN_* and the intracluster data transmission wake-up time slot *T_SIN_*. For each cluster head, it will also create an intercluster TDMA scheduling sequence, calculate the intracluster data reception wake-up time slot *T_RIN_*, the intercluster data reception wake-up time slot *T_ROUT_*, and the intercluster data transmission time slot *T_SOUT_*.

Step 4: In the data collection phase within the cluster, each node wakes up at its *T_RIN_*, starts to receive data and enters sleep after completing all data reception. Each non-cluster head node wakes up again at its *T_SIN_*, performs data aggregation, sends data to the next relay node or the cluster head, and then goes to sleep again.

Step 5: During the data transmission phase between cluster heads, each cluster head wakes up at its *T_ROUT_*, starts to receive data from other cluster heads and enters sleep after completing all data reception. Each cluster head wakes up again at its *T_SOUT_*, performs data aggregation and sends fused data to the next relay cluster head or the BS, and then goes to sleep again.

Step 6: The BS receives data from cluster heads.

Step 7: Each node cyclically performs data reception and data transmission according to its TDMA scheduling order until the end of the current round, and then the process is restarted from step 1.

## 5. Performance Evaluation

### 5.1. Basic Description of Simulation

The simulation tool NS2 is applied to analyze and evaluate network performance and the energy dissipation of the proposed LEACH-CMF protocol. In addition, we compared the routing protocols LEACH-C and LEACH. The simulation parameters are shown in Table 1.

To measure the network performance, we used the first node death time (*T_FND_*), half the node death time (*T_HND_*), and all node death time (*T_AND_*) as the evaluation indicators of network life.

### 5.2. Simulation and Results Analysis

**Figure 9:**
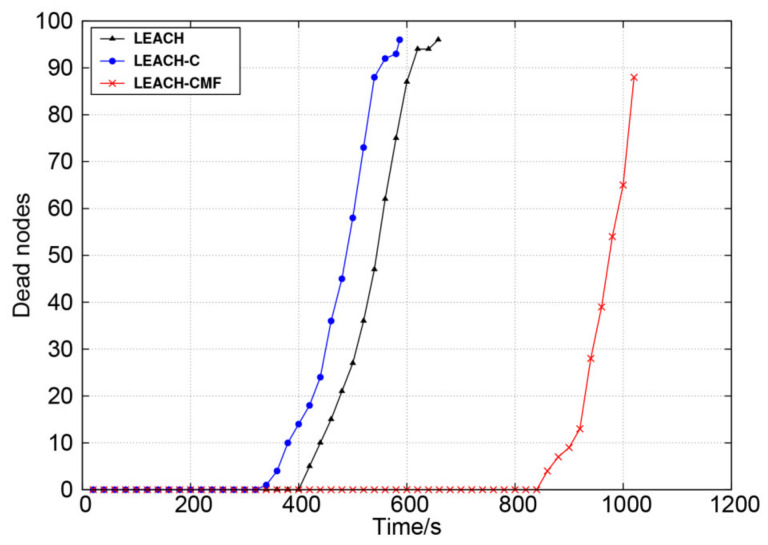
Changes in the number of dead nodes over time.

The network lifetime of the three protocols is shown in Figure 9. It can be seen from the figure that the network lifetime was greatly extended by LEACH-CMF. *T_FND_* was extended by 110% and 160% compared with LEACH and LEACH-C, respectively. Compared with LEACH and LEACH-C, *T_HND_* was extended by 110% and 100%, respectively. The network lifetime was more than twice of LEACH-C. This is because LEACH-CMF shortens communication distance between nodes and cluster heads through intracluster multi-hop communication, and intercluster multi-hop shortens the communication distance between cluster heads and BS, thus slows down the death of the cluster heads to balance the network load.

**Figure 10:**
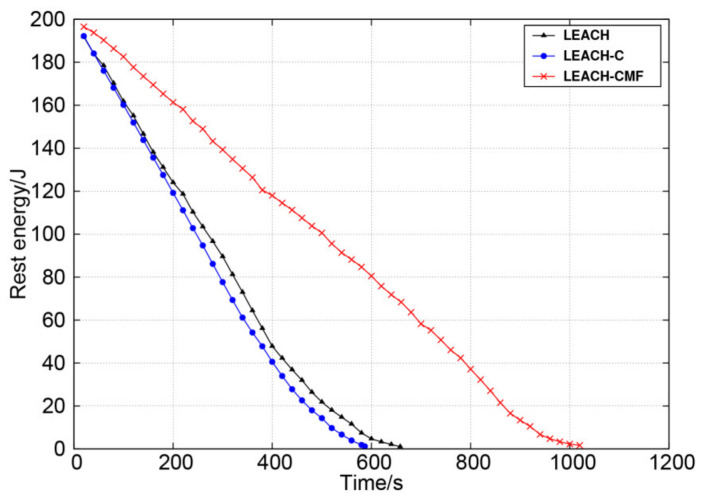
Change of total residual energy over time.

Figure 10 shows the change of the total residual energy. From this figure, it can be observed obviously that LEACH-CMF had better performance. The residual energy of LEACH and LEACH-C dropped significantly more sharply, which means that the two protocols consumed more energy in the routing process. The energy dissipation of LEACH-CMF occurred more slowly than the other two methods. LEACH-CMF greatly shortened the communication distance and achieved the purpose of reducing energy consumption.

**Figure 11:**
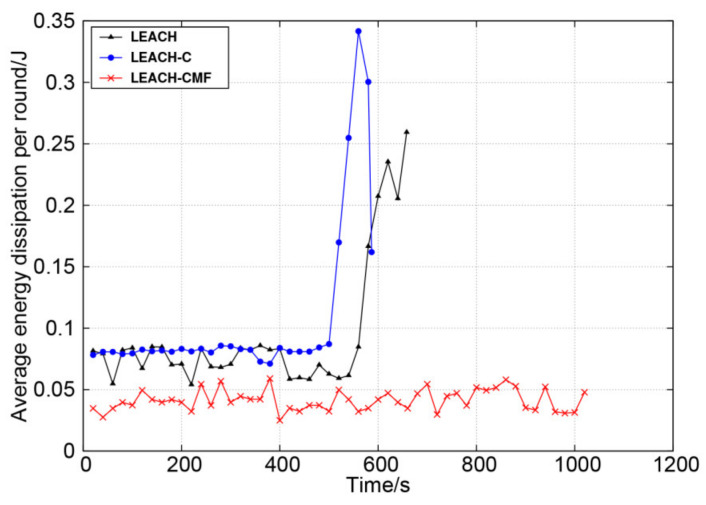
The average node energy dissipation of each round.

Figure 11 shows the change of average node energy dissipation for each round. As can be seen from the figure, LEACH and LEACH-C consumed more energy each round and the increase was obvious at the later stage, reflecting the unstable network performance. LEACH-CMF maintained long-term low energy dissipation and the overall change was small, reflecting stable network performance. Obviously, LEACH-CMF balanced network load efficiently.

**Figure 12:**
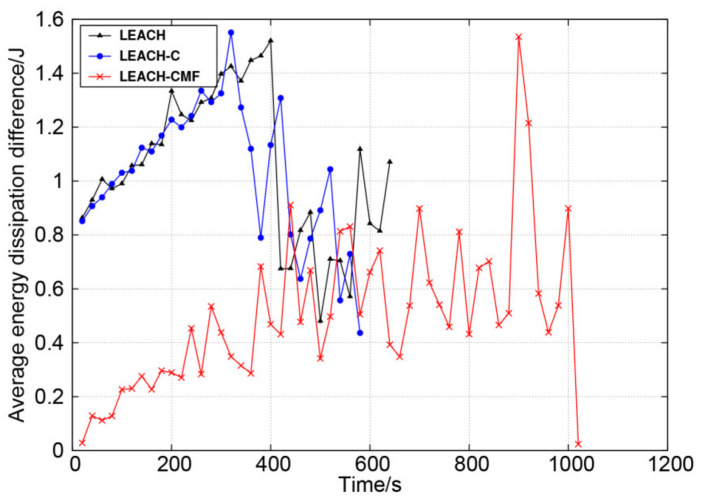
The difference of average energy dissipation per round between cluster heads and non-cluster nodes.

Figure 12 shows the difference of the average energy dissipation per round of cluster heads and the average energy dissipation per round of all nodes. The proposed method had relatively small energy difference, which well balanced the energy dissipation between cluster heads and non-cluster head nodes. That is because the proposed method balanced the load of the cluster heads, which can also perform appropriate hibernation.

## 6. Conclusions

In WSNs, the multi-hop-based transmission and fusion are effective in reducing redundant data communications, saving the energy of sensor nodes, and enhancing network lifetime. This paper analyzed the energy dissipation of relay transmission and non-relay transmission and proposed an optimized relay cost model to select the optimal relay nodes. In order to reduce the energy dissipation in the relay transmission as much as possible, a multi-start minimum spanning forest algorithm was used to select relay nodes and simultaneously generate the network. In order to realize multi-hop transmission with data aggregation, reducing data collision and make nodes sleep as much as possible in the non-working state, we designed a bottom-up continuous time slot allocation method and modified the TDMA cycle. The data sending frequency of different cluster heads at the intercluster transmission stage were matched at the lowest delay, and the cluster heads could also sleep properly in the non-working time.

The simulation results show that the proposed clustering scheme performed better as compared with the other two methods. It prolonged network lifetime and maintained a long-term, small-scale energy dissipation fluctuations, which reflected stable network performance.

## Figures and Tables

**Table 1:** Simulation Parameters.

Parameters	Value
Free space amplification factor $\varepsilon_{fs}$	10 pJ/bit/m^2^
Multipath attenuation amplification factor	0.0013 pJ/bit/m^4^
Data fusion energy dissipation coefficient $E_{da}$	5 nJ/bit
Circuit transmit and receive energy $E_{elec}$	50 nJ/bit
Transmission bandwidth	1 Mbps
Transmission data per frame	500 bytes
The time of a round	20 s
Simulation area of the network *S*	100 m × 100 m
Node initial energy *E_0_*	2 J
Total number of nodes *N*	100
Cluster head ratio *p*	5%
Base station location	(50, 50) m

## Data Availability

No new data were created or analyzed in this study. Data sharing is not applicable to this article.
